# Gamma radiation at a human relevant low dose rate is genotoxic in mice

**DOI:** 10.1038/srep32977

**Published:** 2016-09-06

**Authors:** Anne Graupner, Dag M. Eide, Christine Instanes, Jill M. Andersen, Dag A. Brede, Stephen D. Dertinger, Ole C. Lind, Anicke Brandt-Kjelsen, Hans Bjerke, Brit Salbu, Deborah Oughton, Gunnar Brunborg, Ann K. Olsen

**Affiliations:** 1 Department of Chemicals and Radiation, Norwegian Institute of Public Health, Oslo 0403, Norway; 2 Centre for Environmental Radioactivity (CoE CERAD), Ås 1432, Norway; 3 Department of Environmental Sciences (IMV), Norwegian University of Life Sciences (NMBU), Centre for Environmental Radioactivity (CoE CERAD), Ås 1432, Norway; 4 Litron Laboratories, Rochester, NY 14623, United States; 5 Department of Monitoring and Research, Norwegian Radiation Protection Authority, Østerås 1332, Norway

## Abstract

Even today, 70 years after Hiroshima and accidents like in Chernobyl and Fukushima, we still have limited knowledge about the health effects of low dose rate (LDR) radiation. Despite their human relevance after occupational and accidental exposure, only few animal studies on the genotoxic effects of chronic LDR radiation have been performed. Selenium (Se) is involved in oxidative stress defence, protecting DNA and other biomolecules from reactive oxygen species (ROS). It is hypothesised that Se deficiency, as it occurs in several parts of the world, may aggravate harmful effects of ROS-inducing stressors such as ionising radiation. We performed a study in the newly established LDR-facility *Figaro* on the combined effects of Se deprivation and LDR γ exposure in DNA repair knockout mice (*Ogg1*^−/−^) and control animals (*Ogg1*^+/−^). Genotoxic effects were seen after continuous radiation (1.4 mGy/h) for 45 days. Chromosomal damage (micronucleus), phenotypic mutations (*Pig-a* gene mutation of RBC^CD24−^) and DNA lesions (single strand breaks/alkali labile sites) were significantly increased in blood cells of irradiated animals, covering three types of genotoxic activity. This study demonstrates that chronic LDR γ radiation is genotoxic in an exposure scenario realistic for humans, supporting the hypothesis that even LDR γ radiation may induce cancer.

Humans are exposed to ionising radiation from many sources, including naturally occurring radionuclides (cosmic and terrestrial, e.g. radon gas), certain occupations (e.g., cardiologists and power plant workers), various diagnostic tests and medical therapies (e.g., x-rays or radiotherapy) as well as artificially produced radionuclides released to the environment following nuclear accidents (e.g., reactor accidents in Chernobyl, 1986, and Fukushima, 2011). The risk management of radiation protection of humans is based on a model that assumes a linear relationship with no threshold between radiation dose and health risk. This linear no threshold (LNT) model is based on data from high dose/high dose rate experiments and epidemiological studies, such as those of atomic bomb survivors^1^. It is known that acute radiation gives rise to DNA lesions and causes genotoxic effects at high doses. The effect of chronic exposure to low dose rates (LDR) is less clear, despite its relevance for humans. While the LNT model has guided risk assessment for decades, scientists have expressed a number of concerns regarding the applicability of the model in the *low* dose area (reviewed in ref. 2). For long term exposures below a total dose of 100 mGy, increased cancer risks above background rates are difficult to detect in populations. Hence, there are still large uncertainties about the health risks in the low dose area that need to be addressed. One of the controversial assumptions of the LNT model is that it does not take biological defence mechanisms (e.g. DNA repair) into consideration – mechanisms that could conceivably modify the risk of cancer at low doses. Additionally, very little is known about effects of *chronic* exposures at low dose rates, despite their human relevance. The United Nations Scientific Committee on the Effects of Atomic Radiation (UNSCEAR) defined LDR as below 6 mGy/h and low total dose as below 200 mGy (§16 in ref. 3), later revised to 100 mGy in the UNSCEAR 2013 report^4^. Indeed, very few animal studies have investigated the effects of continuous chronic LDR radiation at below 6 mGy/h^5^,^6^,^7^,^8^,^9^, likely due to the limited number of facilities allowing long term *in vivo* exposure at continuous LDR. This challenge was recognised by the European platform MELODI (Multidisciplinary European Low Dose Initiative) dedicated to low dose radiation risk research (http://www.melodi-online.eu/). One main task was to develop infrastructures to facilitate these kinds of experiments (http://www.doremi-noe.net/). In a project supported by DoReMi (Low Dose Research towards Multidisciplinary Integration) we upgraded an exposure facility (*Figaro*, NMBU) which enabled us to perform long term *in vivo* LDR experiments with gene-modified rodents.

Radiation damages DNA either directly (electrons attack DNA) or indirectly (radiolysis of water forming ROS such as H_2_O_2_, O_2_^−^, lipid hydroperoxides). Radiolysis of water is the predominant mechanism at low doses. When this event occurs in close proximity to DNA it will contribute to oxidative stress in form of ROS. However, the antioxidative defence system, such as glutathione peroxidase (GPx), will catalyse reactions reducing H_2_O_2_ or lipid hydroperoxides (reviewed in ref. 10), thus protecting DNA and other biomolecules. Selenium (Se) is an essential trace element that is incorporated into selenoproteins and is crucial for these proteins’ catalytic activity. One of the main functions of selenoproteins is as the antioxidant GPx^11^. One may therefore hypothesise that Se depletion would aggravate harmful effects induced by LDR radiation.

If the organism fails to eliminate ROS it can cause (oxidised) DNA lesions. An impaired repair of oxidised DNA lesions may therefore also aggravate the effects of chronic exposure to radiation. Mice that lack the repair enzyme OGG1 (*Ogg1*^−/−^), which removes 8–oxoguanine^12^, would be expected to accumulate radiation induced lesions and thereby serve as a sensitive model for oxidative stress. To specifically detect oxidised DNA lesions in the present work, enzymatic treatment of nucleoids with formamido pyrimidine DNA glycosylase (Fpg, a bi-functional DNA glycosylase) was used in the alkaline single cell gel electrophoresis (SCGE) assay. Fpg allows detection of a broad range of oxidised DNA lesions (oxidised purines, 8-oxoguanine, formamido pyrimidines, apurinic/apyrimidinic sites)^13^,^14^,^15^.

The aim of the present study was to investigate the genotoxic effects of chronic LDR γ radiation (1.4 mGy/h for 45 days) in blood from mice. Blood is easy to access and represents a convenient compartment to assess radiation-sensitive biomarkers. In addition, two factors that could potentially influence the manifestation of the genotoxic radiation effects were studied: diet (Se deficiency) and genotype (OGG1 deficiency), as well as combinations thereof.

## Results

In order to investigate the potential influence of a low Se intake on the effects of chronic LDR γ irradiation, mice were Se depleted through two generations. At study start, mice received custom-made diets mainly consisting of wheat grown on Norwegian soil which has a natural low Se concentration (low Se diet), or of Se enriched wheat using fertilizer of different Se concentrations^16^ (normal Se diet). Data presented here is from male mice of the second generation. As previously shown in a pilot study^17^, the GPx activity in plasma was significantly reduced in mice on low Se diet compared with those on normal Se diet (3.6 ± 0.3 vs 12.6 ± 3.6 nmol/min/mg, p = 0.018, t-test). Furthermore, two different genotypes (homo- or heterozygotes of *Ogg1*, a DNA repair enzyme removing 8-oxoguanine) were used. The genotype showed no substantial impact on any studied endpoint.

### Concentration of Se in liver

To confirm Se depletion, total Se concentrations in liver were measured. The mean levels were 0.52 (±1.06) and 3.74 (±1.26) mg Se/kg (dry weight) liver for mice fed the low and normal Se diet, respectively (n = 64 for each diet). Thus, the Se concentration in liver was reduced by 86% when mice were given the low Se diet (p < 0.001) compared with mice fed the normal Se diet. One mouse in the low Se diet group had an unexpected high value of Se in liver (8.81 mg/kg) and was excluded from all further analyses.

### Micronuclei (MN) assay

**Figure 1:**
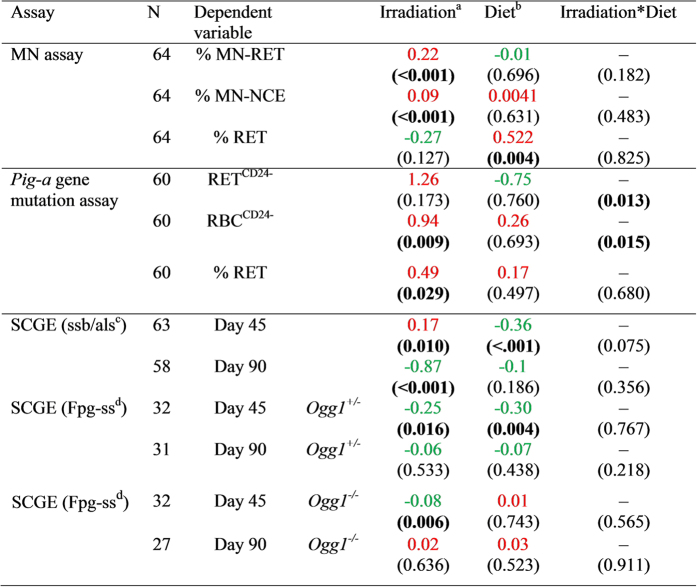
Differences of Least Square Means (LSM) of untransformed data between groups after AIC. cf. *Statistical analysis* in the method section. P-values for the factors irradiation and diet and their interaction (irradiation*diet) are given in brackets (in bold for p < 0.05). LSM are indicated in red for endpoints where irradiation or low Se diet had a stronger effect; LSM are indicated in green for endpoints where non-irradiation or normal Se diet had a stronger effect. ^a^Difference of LSM between irradiated and non-irradiated mice (LSM_IR_ – LSM_nonIR_). Positive values indicate a higher response in the investigated endpoint in the irradiated groups compared with the non-irradiated groups. ^b^Difference of LSM between low and adequate Se diet (LSM_lowSe_ – LSM_normSe_). Positive values indicate a higher response in the investigated endpoint in the groups fed with low Se diet compared with groups fed with normal Se diet. ^c^Single strand breaks and alkali labile sites. ^d^Fpg-sensitive sites, i.e. oxidized DNA lesions.

**Figure 2:**
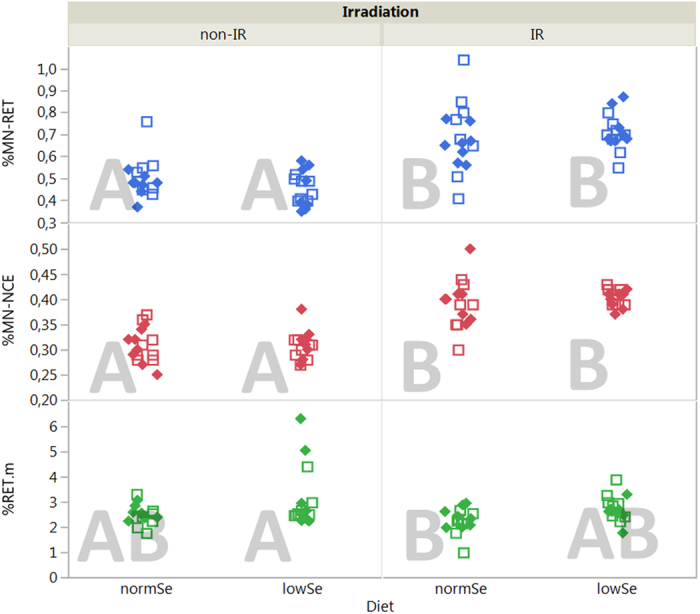
Micronucleus assay. Upper panel: Mean percentage of micronucleated blood reticulocytes (% MN-RET). Middle panel: micronucleated normochromic erythrocytes (% MN-NCE). Lower panel: percentage reticulocytes (% RET) of unexposed (non-IR) and chronic LDR irradiated (IR) mice given two different diets (normSe and lowSe). Solid diamonds (*Ogg1*^+/−^) and hollow squares (*Ogg1*^−/−^) represent individuals (8 mice per group). Similar letters indicate that there is no significant difference between groups (Tukey’s HSD).

Chronic LDR γ radiation induced significantly increased numbers of micronucleated reticulocytes (MN-RET, 40% mean increase) and normochromatic erythrocytes (MN-NCE, 29% mean increase) with p < 0.001 (positive LSM difference in Figs 1 and 2), irrespective of genotype and diet.

The group mean values (for both irradiated and non-irradiated cells) for MN-RBC are more than 50% lower compared with the group mean values for MN-RET (S3). The lower but still significant response in MN-RBCs towards chronic LDR radiation might be explained by the longer lifespan of RBCs (38–55 days, compared with a maximum of five days for RETs, cf. *Study design*) allowing for a higher chance of elimination. Furthermore, some RBCs might have developed from unaffected progenitor cells resulting in a lower absolute effect in MN-RBCs.

Mean percentage of RET were affected by the diet (p = 0.004) with an increase in animals given the low Se diet compared to the normal Se diet (Fig. 1). However, this impact might be caused by only few individuals (Fig. 2) and therefore be of minor significance.

No significant interactions between the three factors radiation, diet, and genotype were identified with respect to MN formation.

### *Pig-a* gene mutation assay

**Figure 3:**
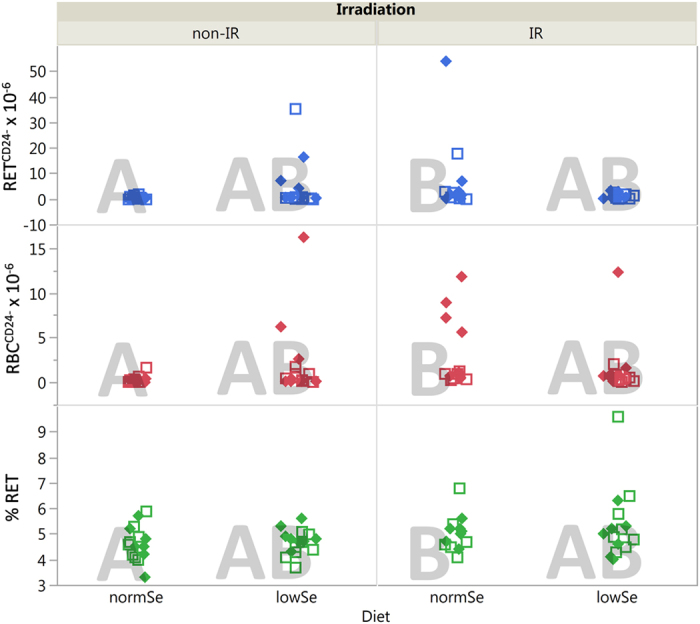
*Pig-a* gene mutation assay. Mutant phenotype frequencies of RET^CD24−^ (upper panel), RBC^CD24−^ (middle panel) and % RET (lower panel) of unexposed (non-IR) and chronic LDR irradiated (IR) mice given two different diets (normSe and lowSe). Solid diamonds (*Ogg1*^+/−^) and hollow squares (*Ogg1*^−/−^) represent individuals (6–8 mice per group). Similar letters indicate that there is no significant difference between groups (Tukey’s HSD).

Data of the *Pig-a* gene mutation assay is presented in Fig. 3. In general, the chronic LDR γ radiation caused a more than two-fold increase in the frequency of mutant phenotypes in erythrocytes (RBC^CD24−^) with p = 0.009 (Fig. 1). There was no significant effect of genotype or diet alone on RBC^CD24−^. Phenotypic mutation frequency of reticulocytes (RET^CD24−^) was not significantly affected by any factor (i.e. irradiation, diet or genotype) alone (Fig. 1).

The *Ogg1*^+/−^ mice showed a higher variation of the mutation frequency of RBC compared with the *Ogg1*^−/−^ mice. However, the possibility that the observations might be due to technical artefacts and/or a chance effect due to the relatively high level of individual variation associated with some mouse models cannot be excluded.

The combination of radiation and diet (IRR*DIE) had an impact on the phenotypic mutation frequency of RETs and RBCs with p = 0.013 and p = 0.015, respectively (Fig. 1).

In both the *Pig-a* gene mutation assay and the MN assay the immature fraction of erythrocytes are identified and scored. The percentage of RET values were generally higher in the *Pig-a* gene mutation assay relative to the MN assay. This finding is related to the manner by which each assay identified and scored the immature fraction of erythrocytes. For the *Pig-a* gene mutation assay, RETs were detected based on their nucleic acid dye-associated fluorescence, whereas the MN assay detected RETs based on cell surface expression of CD71.

Mice treated with ENU (positive assay control; data not shown) showed a significantly higher response in all measured parameters (6.25% RET and a mutant phenotype frequency of 96.1 and 23.75 for RET^CD24−^ and RBC^CD24^, respectively).

### Single cell gel electrophoresis (SCGE) assay

**Figure 4:**
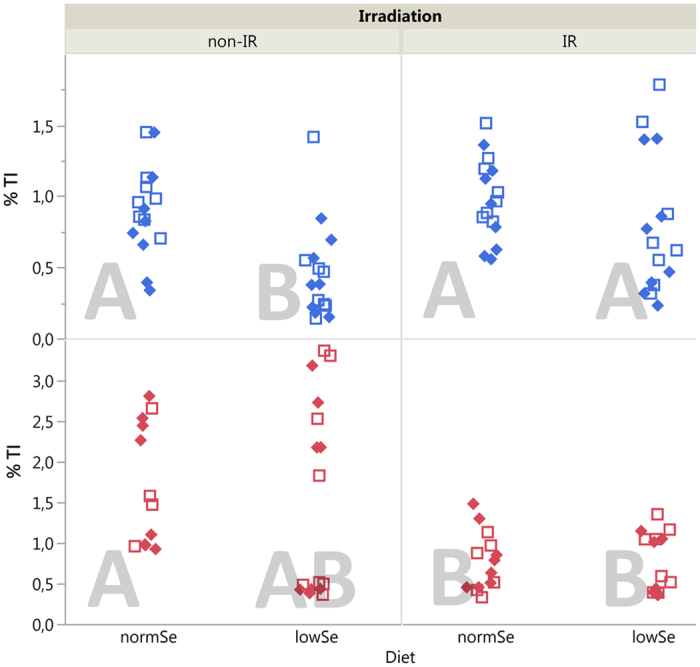
SCGE assay, ssb/als. Single strand breaks and alkali labile sites (ssb/als) detected by the alkaline SCGE assay in whole blood of mice after 45 days (upper panel) of chronic LDR γ radiation and at day 90, i.e. after 45 days recovery (lower panel). Solid diamonds (*Ogg1*^+/−^) and hollow squares (*Ogg1*^−/−^) represent individuals (8 mice per group). Similar letters indicate that there is no significant difference between groups (Tukey’s HSD).

**Figure 5:**
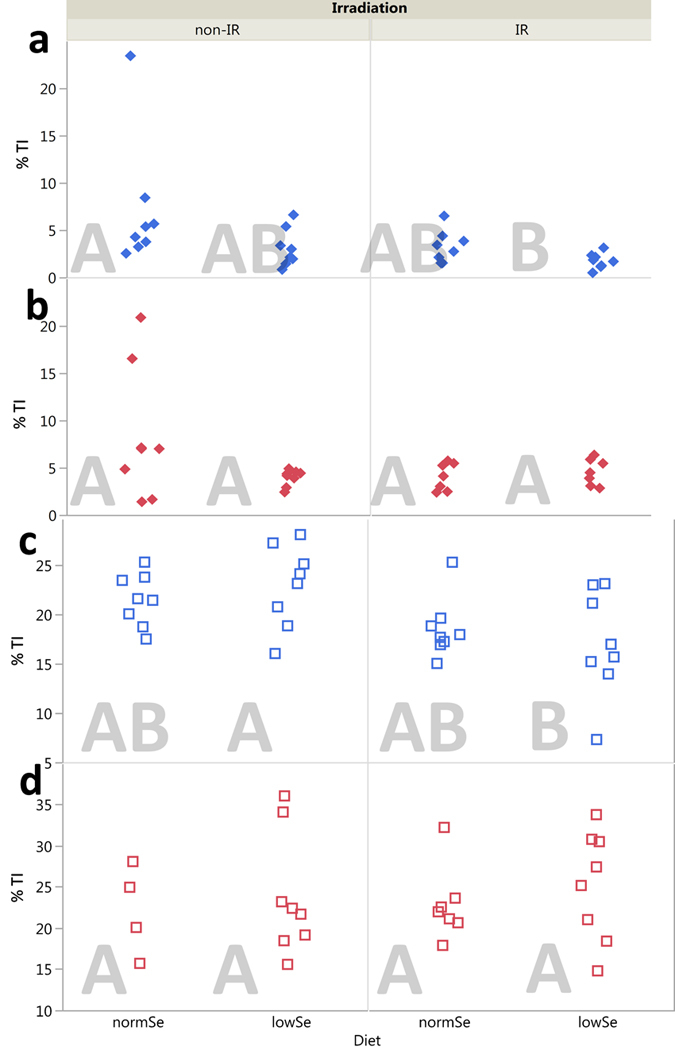
SCGE assay, oxidised DNA lesions. Fpg-sensitive sites (Fpg-ss) detected by the alkaline SCGE assay in whole blood of mice for each time point and genotype separately. Panel A: *Ogg1*^+/−^ at day 45; Panel B: *Ogg1*^+/−^ at day 90; Panel C: *Ogg1*^−/−^ at day 45; Panel D: *Ogg1*^−/−^ at day 90. Solid diamonds (*Ogg1*^+/−^) and hollow squares (*Ogg1*^−/−^) represent individuals (4–8 mice per group). Similar letters indicate that there is no significant difference between groups (Tukey’s HSD).

Data from the SCGE assay is shown in Figs 4 and 5. Figure 4 depicts single strand breaks and alkali labile sites (ssb/als) at day 45 (i.e. blood collected immediately after cessation of radiation) and day 90 (i.e. blood collected 45 days after cessation of radiation). Oxidised DNA lesions (revealed by Fpg, i.e. Fpg-sensitive sites, Fpg-ss) are shown in Fig. 5.

The levels of ssb/als were significantly impacted by diet and radiation at day 45 (Figs 1 and 4). Overall, mice given the low Se diet had almost two-fold lower levels of DNA lesions (ssb/als) than mice given the normal Se diet (89% decrease, p < 0.001, negative LSM difference). Irradiated mice showed higher levels of DNA lesions (ssb/als) compared to non-irradiated mice (p = 0.010, positive LSM difference, Fig. 1). Forty-five days after radiation stop (at day 90), the effect of radiation on the levels of DNA lesions (ssb/als) was inverse compared with day 45 (negative LSM difference, Fig. 1): the levels of ssb/als were a factor of two lower in irradiated mice compared to non-irradiated mice (p = 0.001). This effect was independent of diet and genotype.

Oxidised DNA lesions were analysed separately for *Ogg1*^+/−^ (Fig. 5a,b) and *Ogg1*^−/−^ mice (Fig. 5c,d). The OGG1-deficient mice have a naturally higher background of oxidised DNA lesions that could mask possible effects when analysing both genotypes together. In *Ogg1*^+/−^ mice, both radiation and diet had a significant impact on the Fpg-ss (p = 0.016 and p = 0.004, respectively). The most stressed group (low Se diet and radiation) had less oxidised DNA lesions compared with the control group (normal Se diet and no irradiation), p < 0.05. Knockout mice were not affected by Se in the diet, while radiation reduced the level of oxidised DNA lesions, p = 0.006 (Figs 1 and 5c,d).

Oxidised DNA lesions (Fpg-ss) assessed at day 90 were not affected by diet or radiation, neither in *Ogg1*^+/−^ nor *Ogg1*^−/−^ (Figs 1 and 5b,d).

As expected, oxidised DNA lesions at both sampling time points were dependent on the genotype, with a significant higher level of DNA lesions in *Ogg1*^−/−^ compared with *Ogg1*^+/−^ mice.

Detailed data for each endpoint (Micronuclei assay, *Pig-a* gene mutation assay, SCGE assay) is given in the supplementary material (S1-3).

## Discussion

To date, only few studies have addressed the effects of LDR radiation within a range which is of human relevance as it can be found after accidents like in Chernobyl and Fukushima. Even fewer have investigated the biological effects of continuous *chronic* LDR radiation despite their relevance in occupational and environmental contamination settings. This is likely due to the limited number of animal facilities allowing long term *in vivo* exposure to IR. Studies on genotoxic effects caused by Se depletion starting *in utero* are also scarce. Due to the central role of Se in the antioxidative defence system (Gpx), low Se levels may increase the susceptibility to radiation-induced ROS damaging the DNA or other macro-molecules. The current study is the first mouse study conducted in the newly established γ exposure facility *Figaro* where continuous and chronic low doses can be explored. To our knowledge this is also the first study to investigate genotoxic effects on the combined stressors LDR γ radiation and Se deficiency. In the following, the effects of the factors radiation, diet, and genotype are discussed separately prior to a discussion of their combined effects.

In the present study we demonstrate that exposure to a human relevant LDR γ radiation induces genotoxic effects in mouse blood cells assessed with three separate but complementary assays. These effects were expressed as increased levels of chromosomal damage (micronuclei), phenotypic mutations (RBC^CD24−^) and DNA lesions (ssb/als). The absolute measured changes were small, but significant. The formation of MN was observed in all irradiated groups independent of genotype or diet, and significant changes were seen in both immature and mature erythrocytes. This is an expected result given the chronic exposure and lack of splenic filtration of circulating MN-containing erythrocytes^18^.

Existing data on mutagenicity of LDR radiation is limited and the results in the literature are not consistent. Osipov and colleagues reported a genotoxic effect following very LDR exposure of mice with increased MN frequency in bone marrow cells and increased levels of ssb/als in spleen cells, applying a 20-fold lower dose rate (0.07 mGy/h) and 7-fold lower accumulated dose (70–200 mGy) compared to the present study design^9^. In contrast, Olipitz and colleagues did not observe increased levels of base lesions in spleen cells or micronucleated erythrocytes (applying a dose rate of 0.102 mGy/h and total dose of 105 mGy)^5^. Relative to the two above-mentioned reports which used microscopic analysis to detect MN, the current study benefitted from automated scoring using a flow cytometer. This facilitated objective analysis of 20,000 reticulocytes and hundreds of thousands of erythrocytes per mouse (compared to some thousand cells using the microscopic analysis), thereby providing greater statistical power to detect weak effects of the magnitude described herein. Wickliffe and colleagues exposed BigBlue^®^ C57BL/6 hemizygous mice in the Red Forest area of Chernobyl, achieving the same LDR as in the present study (1.4 mGy/h)^6^. Even though the exposure time was twice as long as that of our study (90 days of irradiation), no increased levels of mutation frequencies were detected with the conventional *lacI* transgenic rodent mutation assay. However, the number of putative mutant clones assessed was low, reducing the possibility of identifying a genotoxic effect. In our study, we assessed mutations by the *Pig-a* gene mutation assay based on flow cytometry. There are only few studies exploring the mutagenic effect of ionising radiation by the *Pig-a* gene mutation assay^19^,^20^,^21^ applying acute exposure with x-rays, and not chronic exposure with LDR γ-rays as done in the present study. Significant increases of mutation frequencies were detected in RET^CD24−^ and RBC^CD24−^ after 1 Gy total dose (C57BL/6J mice)^20^,^21^ and in RBC ^CD24−^ after 2 Gy total dose (F344 rats)^19^ of acute x-ray exposure.

It has been estimated that one Gy of γ radiation as used herein causes 1000 ssb, 500 base damages, 40 double strand breaks (dsb) and 150 DNA protein cross-links in one mammalian cell (p. 351 in ref. 22). Based on this value and a simplistic linear extrapolation, our applied dose rate of 1.4 mGy/h would be expected to cause approximately 34 ssb per cell per day. This seems to be a negligible amount compared to the approximately 200,000 spontaneously induced ssb a mammalian cell has to cope with every day^23^. However, radiation-induced DNA lesions are more complex than endogenous DNA lesions^24^ and can therefore cause an overload of the natural repair capacity and potentially become deleterious over time^25^. Ionising radiation also produces free radicals that can cause clustered DNA lesions. These types of lesions occur when at least two radical hits occur within 20 base pairs^26^ and are assumed to contribute to the harmful effect of radiation. This is partly due to the compromised repair of such lesions and partly because IR causes a broad spectrum of DNA lesions^27^. The repair process depends strongly on the modified DNA components within the cluster. It has been suggested that dsb may be formed during the repair process of these clusters, contributing to mutagenesis^28^. In fact, in pro- and eukaryotic cells it has been shown that DNA clusters are prone to result in dsb^29^,^30^. Hence, this might explain the increased mutation frequencies that we have observed in some irradiated mice. However, studies on clustered DNA lesions have been using acute and high dose rates of ionising radiation. D. P. Hayes suggests that at a low dose/dose rate (as applied in this study) repair mechanisms might not be activated resulting in elimination of the cell by apoptotic or mitotic death^31^.

Interestingly, at day 90 (45 days after cessation of radiation) the levels of ssb/als in irradiated mice were approximately 50% lower than in non-irradiated mice (Fig. 5). A constant exposure to a stressor (e.g. LDR γ radiation) may not induce DNA repair when the total dose stays below a threshold level, as suggested in *in vitro* systems (eukaryotes)^32^ and in fish^33^. On the other hand, defence mechanisms (e.g. DNA repair mechanisms) might kick in when a certain total dose is approached^34^. Hence, we assume that the observed effect in our study could potentially reflect an induction of a protective response towards irradiation.

Se is an essential trace element which is, for example, incorporated in enzymes (e.g. GPx) as selenoprotein catalysing ROS-eliminating reactions (e.g. reducing H_2_O_2_ and lipid hydroperoxides). An inadequate Se intake was therefore hypothesised to cause additional oxidative stress when combined with LDR γ radiation. Mice were fed a diet made out of locally grown wheat (resulting in Se deficient diet) or wheat fertilized with Se (normal Se diet) for two generations, thus assuring a complete Se depletion. The Se content in the diet had a noteworthy effect on the level of DNA lesions in blood measured at the end of γ exposure in mice (at day 45). The levels of ssb/als and oxidised DNA lesions (i.e. Fpg-sensitive sites, Fpg-ss) were marginal reduced in mice fed the Se deficient diet (p < 0.001 and p = 0.004, respectively). This observation was surprising since Se has a major role in the antioxidative defence system and a raised level of (oxidised) DNA lesions would have been expected. However, measurements of the GPx-activity in plasma (i.e. one part of the antioxidative defence system) were significantly reduced in the groups fed with low Se diet. It is possible that other antioxidant enzymes (e.g. superoxide dismutase and catalase which do not require secondary enzymes such as GPx) are up-regulated to back-up for the missing GPx activity. Even though the reduced levels of DNA lesions in the low Se group were significant, the difference is small (Figs 4 and 5) and should be weighed with care. In men with a low Se blood level elevated levels of DNA lesions (ssb/als) in leukocytes have been observed^35^. We have recently shown that the levels of DNA lesions in lung and testis of Se depleted mice were also elevated^17^. Furthermore, low Se blood levels have been associated with increased risk of cancers such as prostate cancer^36^, lung cancer^37^ and colorectal cancer^38^.

To study the implications of the lack of a DNA repair enzyme, namely DNA glycosylase OGG1, the *Ogg1*^−/−^ genotype was included in this study. The *Ogg1*^−/−^ accumulates oxidised DNA lesions due to its reduced capacity to excise 8-oxoG, a highly premutagenic oxidised base lesion (measurable in the SCGE in combination with the DNA repair enzyme Fpg). Interestingly, the lack of OGG1 had no impact on any studied endpoint in blood. Neither the chronic LDR of γ radiation nor the low Se diet led to additional levels of oxidised DNA lesions in *Ogg1*^−/−^ mice detectable in the SCGE. It is possible that the low dose rate did not produce enough free radicals to induce a detectable level of oxidised DNA lesions. Another explanation might be that repair mechanisms for the induced oxidised DNA lesions may have been sufficiently active or up-regulated to compensate for radiation- or diet-induced DNA lesions. For instance, other DNA glycosylases besides OGG1 exist in mammalian cells in the base excision repair (BER) pathway (e.g., MYH, NEIL1, NEIL2, NEIL3 and NTH1). BER is the major DNA repair pathway for oxidised DNA lesions induced by ionising radiation^39^,^40^. Similar to OGG1, MYH prevents G to T mutations by removing misincorporated adenine opposite of 8-oxoG^41^. Thus, MYH might mask possible effects of LDR γ radiation.

The next issue was to identify potential interactions between the different stressors radiation, diet, and genotype. In general, there are few *in vivo* studies on the combined effects of Se and γ radiation. The few conducted studies have investigated the potential radio-protective character of Se supplementation when applying high doses and dose rates of irradiation (acute, not chronic)^42^,^43^. Studies on the consequences of Se deficiency in conjunction with realistic exposure to chronic LDR irradiation are missing, as well as studies on the genotoxic effect of these combinations. LDR γ radiation combined with low Se diet was hypothesised to give rise to additive or synergistic effects, and thus the opposite of the observed antagonistic effects in this study. Decreased levels of oxidised DNA lesions were measured in mice of the most stressed group, i.e. depleted of Se and subjected to γ radiation (Fig. 5), despite the fact that Se acts as co-factor for GPx. The *Ogg1*^+/−^ mice showed a 40–50% reduced level of oxidised DNA lesions dependent on radiation and diet, with the lowest level in irradiated mice on low Se diet (at day 45) (Fig. 5a). Similar to observations of DNA breaks, an interaction between radiation and diet was also evident for phenotypic *Pig-a* gene mutation frequencies (Figs 1 and 3). The data proposes that defence mechanisms (such as antioxidant systems and repair of oxidised DNA lesions) might be triggered by the combined stressors (Se deficiency and γ radiation) to prevent the induction of harmful effects.

This study contributes with valuable data concerning genotoxic effects of chronic LDR γ radiation alone and in combination with another stressor, low Se diet. The observed changes are small, as expected for experiments using low doses and dose rates of exposure, but the applied doses are realistic in a human relevant context. Nevertheless, significant differences were identified supporting the high sensitivity for the assays, as shown previously for the SCGE assay^44^. We have found that chronic LDR γ radiation induces genotoxic effects in murine blood cells using three assays covering different endpoints (chromosomal fragmentations, phenotypic *Pig-a* gene mutations and DNA lesions). Data suggests that Se depletion partly influences the genotoxic effects of chronic LDR γ radiation with respect to levels of DNA lesions and phenotypic *Pig-a* gene mutation frequencies. A low Se diet seemed to moderate the levels of DNA lesions induced by γ radiation independently of the presence of DNA glycosylase OGG1. Furthermore, prolonged LDR γ radiation appeared to induce a protective response, with lower levels of ssb/als in irradiated mice compared with concurrent non-irradiated mice.

In summary, exposure to chronic LDR of ionising radiation is indeed genotoxic with potential implications for cancer development, and the response is modified by the availability of Se, an element involved in the antioxidative defence system.

## Methods

### Reagents, kits, and feed components

Lympholyte^®^-Mammal cell separation reagent was from CedarLane, Burlington, ON, Canada. Anti-PE MicroBeads, LS+ Positive Selection Columns and QuadroMACS™ Separator were from Miltenyi Biotec GmbH, Bergisch Gladbach, Germany. CountBright™ Absolute Counting Beads were from Invitrogen, Life Technologies™, Carlsbad, CA, USA. Heat-inactivated foetal bovine serum (FBS) was from PAA Laboratories, Pasching, Austria. Anticoagulant Solution, Buffered Salt Solution, Nucleic Acid Dye Solution (SYTO^®^13), Anti-CD24-PE and Anti-CD61-PE were from the Prototype Mouse MutaFlow^®^ kit. This kit and the micronucleus analysis kit (*In Vivo* Mouse MicroFlow^®^ Basic) were from Litron Laboratories, Rochester, NY, USA. N-Nitroso-N-Ethylurea (ENU, cat. no. 3385) was from Sigma Aldrich Norway AS, Oslo, Norway. Low melting point agarose (NuSieve^®^GTG^®^Agarose) and Gelbond^®^ films were from Lonza, Rockland, ME, USA. SYBR^®^Gold Nucleic Acid Gel Stain (10,000× concentrate in DMSO) was from Life Technologies™, Carlsbad, CA, USA. DL-Methionine (cat. no. CA.10850), Torula Yeast Lake States (cat. no. 2184-55), Mineral Mix with Se omitted (cat. no. TD.80313) and Vitamin Mix (cat. no. CA.40060) were from Harlan Laboratories, Madison, WI, USA. Corn oil, calcium carbonate (CaCO_3_) and sucrose were from Fôrtek, Center for Feed Technology, Aas, Norway.

### Animals

**Figure 6:**
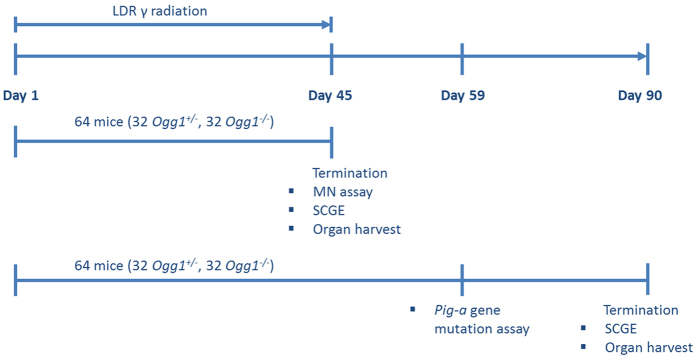
Study design with respect to irradiation and endpoint analysis. The mice were continuously exposed to LDR γ radiation for 45 days. At day 45, blood samples for MN assay and SCGE were taken from 64 mice before killing and harvest of organs. The remaining 64 mice were kept for another 45 days without irradiation (recovery). Blood samples for *Pig-a* gene mutation assay and SCGE were taken on day 59 and day 90, respectively. These mice were killed at day 90 and organs were harvested.

C57BL/6N mice with either OGG1 heterozygote genotype (*Ogg1*^+/−^, phenotypical “wildtype”) or OGG1 knockout genotype (*Ogg1*^−/−^) were bred in-house as described previously^17^. The study included 128 mice (nominally eight mice per group for two different time points; see *Study Design* and Fig. 6). Littermates of both genotypes were housed in each cage.

Mice were acclimated for one week in the NMBU irradiation facility *Figaro* prior to irradiation. Mice were kept at a 12 h light/dark cycle and controlled temperature (22–24 °C) and humidity (55 ± 10%) using the Scantainer/Scanclime system (Scanbur Technology, Karlslunde, Denmark), in accordance with the European convention (2006), appendix A. At study start (i.e. start of exposure to irradiation at day 1) mice were 5–16 weeks old, had an average bodyweight of 25.2 g (18.2 g; 31.9 g) and were randomly assigned to irradiation/non-irradiation group. Two to five mice were kept per disposable PET plastic cage (Innovive, San Diego, CA, USA) with aspen bedding (Nestpack, Datesand Ltd, Manchester, UK).

Two additional mice were treated with ENU (ip injection, 40 mg/kg bw on three consecutive days, total dose of 120 mg/kg bw) and used as positive controls in the *Pig-a* gene mutation assay. The experiments were performed in conformity with the laws and regulations for animal experiments in Norway and were approved by the Norwegian Animal Research Authority.

### Diet

To study the effect of Se deficiency, mice were kept on Se depleted feed over two generations. Thus, offspring used in this study were exposed to Se deficiency *in utero* as described previously^17^: Mice (P) were randomly chosen for either normal (0.23 mg Se/kg diet, Harlan Laboratories, HT2019) or low Se diet (0.01 mg Se/kg diet, Harlan Teklad, TD92163). Only male mice from the second (F1.2) or later litter were allocated to this study. At study start mice were fed a custom-made feed with equal Se-concentrations as the commercial diets (Table 1). The diets, produced at Fôrtek (Aas, Norway), were based on wheat grown locally on a research field (deficient Se diet) and enriched with Se using fertilizer of different Se concentrations^16^ (sufficient Se diet). Forage and Se free water (type I water acidified with 2 mM HCl to prevent bacterial growth) were available for all mice *ad libitum* throughout the study.

### Chronic low dose rate γ radiation (45 days)

Mice were continuously exposed to γ radiation for 45 days from a ^60^Co source (450 GBq) at the NMBU *Figaro* facility. The absorbed dose rate at different distances from the source along the beam axis is available^45^. The continuous irradiation was interrupted on a daily basis for approximately two hours for animal care purposes. Cages were daily moved one position to the right in the rack and irradiated with 1.41 mGy/h (0.99–1.73 mGy/h) giving a total absorbed dose to water of 1.48 Gy (1.04–1.82 Gy). The range of dose rates and total doses was estimated using a phantom mouse (50 ml tube filled with 10% (w/v) gelatine) taking into account all possible positions of the mouse in the cage. The uncertainty of the dose estimates was 10% (95% confidence level). Total doses were controlled using two types of dosimeter systems applied to selected cages: TL-dosimeters (SCK•CEN, Mol, Belgium) and alanine dosimeters (National Physical Laboratory, Teddington, UK). Non-irradiated mice were kept in the same room, but outside of the irradiation field in separate Scantainers. Due to scattered radiation in the room, the control mice were exposed to a γ dose rate of 0.002 mGy/h (total dose of 0.00189 Gy).

### Study design

The study was designed as 2^3^ factorial to investigate the factors irradiation, diet, genotype, and interactions between these factors. Each factor had two levels (irradiated (IR) and non-irradiated (non-IR); normal Se diet (normSe) and low Se diet (lowSe); heterozygote (*Ogg1*^+/−^) and knockout (*Ogg1*^−/−^)) resulting in eight groups. Half of the mice (n = 64) were continuously irradiated for 45 days (starting at day 1 until day 45) (Fig. 6). The duration of the exposure was chosen to mimic a chronic exposure scenario. After this period, the majority of erythrocytes isolated for analysis originate from irradiated erythroid progenitor cells (duration of haematopoiesis in mice is 38–55 days^46^,^47^,^48^,^49^ in which reticulocytes mature for four to five days with limited release into the circulation (about 2–5%^50^).

The data presented here focuses on genotoxic effects in blood. Half of the mice were killed by cervical dislocation at day 45 and the other half at day 90 (after 45 days “recovery period”) to harvest organs (data to be presented elsewhere). Blood samples for the MN assay and the alkaline SCGE assay were taken at the end of irradiation (at day 45), whereas blood for the *Pig-a* gene mutation assay was taken two weeks after cessation of irradiation (at day 59). Forty-five days after irradiation stop (at day 90) additional blood samples were taken for the SCGE assay. Data are from the same animals for the MN assay and SCGE assay (day 45) and for the *Pig-a* gene mutation assay (day 59) and SCGE (day 90), respectively.

### Blood sampling

Blood samples were taken from the saphenous vein using a 21-G needle and a heparinised capillary tube (Bilbate Ltd, Daventry, UK). To assure free-flowing blood, mice were pre-warmed under a heating lamp. For the MN assay, 60 μl of free-flowing blood were added to 350 μl anticoagulant (supplied with the MicroFlowBASIC, Litron Laboratories), mixed well, and kept at room temperature (RT). For the SCGE assay 30 μl of free-flowing blood were added to 100 μl anticoagulant, mixed well, and kept on ice. For the *Pig-a* gene mutation assay 60 μl of free-flowing blood were added to 100 μl anticoagulant, mixed well, and kept at RT. Further processing of all blood samples was performed within two hours.

### Se measurements

Liver samples (about 100 mg tissue) were freeze dried and digested as described previously^17^. Bovine Liver (NIST 1577 b) and Wheat flour (NIST 1567 b) were used as certified reference material in the Se measurements. Indium and tellurium were used as an internal standard correcting for loss during sample preparation, sample introduction, and possible matrix effects on the ICP-MS.

### Micronucleus (MN) assay

Diluted blood samples (60 μl blood + 350 μl anticoagulant) were fixed in ultra-cold pure methanol for ten days and transferred to Long-Term Storage Solution (provided in the kit) as described in the *In Vivo* MicroFlow^®^ Basic instruction manual (version120702). Further processing and analysis of the samples was performed by Litron Laboratories and has been described elsewhere^51^. Malaria-infected erythrocytes served as a biological standard to calibrate the instrument. Approximately 20,000 CD71-positive RETs per sample were acquired on a flow cytometer running CellQuest Pro v5.2 (Becton Dickinson, San Jose, CA, USA).

### *Pig-a* gene mutation assay

The *Pig-a* gene mutation assay was performed as previously described^52^ and in the Prototype Mouse MutaFlow^®^ instruction manual (version130131). On average 8 × 10^6^ RETs and 169 × 10^6^ RBCs per sample were analysed.

It should be noted that the Prototype Mouse MutaFlow^®^ protocol as used herein has been modified since this study was completed. In the current protocol (version140422), post-column samples are no longer incubated at 37 °C, a step that is now known to lyse mouse erythrocytes, especially RNA-negative erythrocytes. It is therefore likely that the RBC^CD24−^ values presented herein may underestimate treatment-induced effects.

For technical reasons, two mice were excluded from the *Pig-a* analysis (wildtype, non-IR, normSe and knockout, IR, normSe; data not shown).

### Single Cell Gel Electrophoresis (SCGE) assay

A high throughput of the alkaline SCGE version^44^ was used, with minor modifications, and has been described in detail more recently^52^. Blinded scoring of the gels was performed. Some samples of knockout mice (*Ogg1*^−/−^) gave an unusual low level of oxidised DNA lesions (Fpg-ss, Fig. 5). Samples that gave levels of DNA lesions below 10% TI (tail intensity, i.e. % tail DNA) were excluded from analysis (n = 5) based on our experience with response in knockout mice after Fpg-treatment in the SCGE assay in blood cells^52^,^53^.

### Statistical analysis

The data for the *Pig-a* gene mutation assay was processed and calculated as described previously^54^ using Microsoft Excel 2010. The raw data of the SCGE was processed using the Comet Assay Spreadsheet Generator Version 1.3.1 (Perceptive Instruments Ltd). The % TI of 50 comets were summarised as median (per gel) and three gels per animal (technical replicates) were summarised as mean, as suggested by Bright *et al*.^55^.

Further statistical analysis was performed using JMP Pro 12 (Statistical Analysis System Institute Inc., Cary, NC, USA). Data from the *Pig-a* gene mutation assay and SCGE assay were log_10_-transformed to achieve the best fit of normal distribution of residuals. An offset of 0.1 was added to values of RET^CD24−^ and RBC^CD24−^ (*Pig-a* gene mutation assay) prior to transformation.

The three factorial (2^3^) study design demanded analysis by a three-way analysis of variance (ANOVA) to identify factors of significant impact, i.e. irradiation, diet or genotype or an interaction of these factors. AIC (Akaike’s information criteria) was used to decide the best model fit, i.e. identify factors that are of no significant impact to the model.

LSMeans (least squares means) Tukey’s HSD (honestly significant difference) was applied to test for differences between groups (α = 0.05). Groups that are significantly different from each other are marked with different letters in the corresponding plots (Figs 2,3,4 and 5).

Due to their genotype, *Ogg1*-knockout mice have a natural higher response after Fpg treatment in the SCGE assay. To avoid an artificial impact of this treatment, genotypes were analysed separately for the SCGE data, i.e. a two way ANOVA was exercised (Fig. 5).

## Additional Information

**How to cite this article**: Graupner, A. *et al*. Gamma radiation at a human relevant low dose rate is genotoxic in mice. *Sci. Rep.* **6**, 32977; doi: 10.1038/srep32977 (2016).

## Supplementary Material

Supplementary Information

## Figures and Tables

**Table 1:** Composition of Se diets.

	% weight		
	Deficient Se diet (0.01 ppm)	Sufficient Se diet (0.23 ppm)	Supplier^a^
Wheat (0.0154 mg Se/kg)	70	57.4	NMBU
Wheat (1.74 mg Se/kg)	—	12.6	NMBU
DL-Methionine (CA.10850)	0.2	0.2	Harlan
Torula Yeast, Lake States (Code 2184-55)	13.5	13.5	Harlan
Corn Oil	5.0	5.0	Fôrtek
Mineral Mix with Selenium Omitted (TD.80313)	3.5	3.5	Harlan
CaCO_3_	1.1	1.1	Fôrtek
Vitamin Mix, Teklad (CA.40060)	1.0	1.0	Harlan
Sucrose	5.7	5.7	Fôrtek

^a^NMBU, Norwegian University of Life Science, Aas, Norway; Fôrtek, Center for Feed Technology, Aas, Norway; Harlan Laboratories, Madison, WI, USA.
