# Supplementary material for: Gamma radiation at a human relevant low dose rate is genotoxic in mice
Source: Sci Rep. 2016 Sep 6;6:32977. doi: 10.1038/srep32977 (PMC5011728; doi:10.1038/srep32977)
Supplement: Supplementary Information [file srep32977-s1.pdf]

Gamma radiation at a human relevant low dose rate is genotoxic in mice

Anne Graupner[1,5,*], Dag M. Eide[1,5], Christine Instanes[1,5], Jill M. Andersen[1,5], Dag A. Brede[2,5], Stephen D. Dertinger[3], Ole C. Lind[2,5], Anicke Brandt-Kjelsen[2], Hans Bjerke[4], Brit Salbu[2,5], Deborah Oughton[2,5], Gunnar Brunborg[1,5], Ann K. Olsen[1,5]

S1. Original data for the measurement of DNA lesions by the Single Cell Gel Electrophoresis.

				Ssb/als [%TI]			Oxidised DNA lesions [%TI]		
Irradiation	Diet	Genotype	Experimental Day	N	Mean	SD	N	Mean	SD
non-IR	normSe	*Ogg1*$^{+/-}$	Day 45	8	0.8	0.4	8	7.1	6.8
non-IR	normSe	*Ogg1*$^{-/-}$	Day 45	8	1.0	0.2	8	21.5	2.6
non-IR	lowSe	*Ogg1*$^{+/-}$	Day 45	8	0.4	0.3	8	3.1	2.0
non-IR	lowSe	*Ogg1*$^{-/-}$	Day 45	8	0.5	0.4	8	23.0	4.1
IR	normSe	*Ogg1*$^{+/-}$	Day 45	8	0.9	0.3	8	3.2	1.7
IR	normSe	*Ogg1*$^{-/-}$	Day 45	8	1.1	0.2	8	18.6	3.0
IR	lowSe	*Ogg1*$^{+/-}$	Day 45	8	0.7	0.5	8	1.7	0.8
IR	lowSe	*Ogg1*$^{-/-}$	Day 45	8	0.8	0.5	8	17.1	5.3
non-IR	normSe	*Ogg1*$^{+/-}$	Day 90	8	1.8	0.8	8	8.3	6.9
non-IR	normSe	*Ogg1*$^{-/-}$	Day 90	4	1.7	0.7	4	22.3	5.4
non-IR	lowSe	*Ogg1*$^{+/-}$	Day 90	8	1.5	1.2	8	3.9	0.9
non-IR	lowSe	*Ogg1*$^{-/-}$	Day 90	8	1.6	1.3	8	23.9	7.4
IR	normSe	*Ogg1*$^{+/-}$	Day 90	8	0.8	0.4	8	4.2	1.4
IR	normSe	*Ogg1*$^{-/-}$	Day 90	7	0.8	0.3	7	22.9	4.5
IR	lowSe	*Ogg1*$^{+/-}$	Day 90	7	0.7	0.4	7	4.6	1.4
IR	lowSe	*Ogg1*$^{-/-}$	Day 90	8	0.8	0.4	8	25.3	6.7

S2. Original data for the measurement of phenotypic mutations by the *Pig-a* gene mutation assay.

				RET^{CD24-}			RBC^{CD24-}			% RET		
Irradiation	Diet	Genotype	Experimental Day	N	Mean	SD	N	Mean	SD	N	Mean	SD
non-IR	normSe	*Ogg1*$^{+/-}$	Day 59	7	0.3	0.3	7	0.2	0.1	7	4.6	0.8
non-IR	normSe	*Ogg1*$^{-/-}$	Day 59	8	0.7	0.8	8	0.5	0.5	8	4.7	0.6
non-IR	lowSe	*Ogg1*$^{+/-}$	Day 59	8	3.7	5.7	8	3.2	5.7	8	4.9	0.4
non-IR	lowSe	*Ogg1*$^{-/-}$	Day 59	8	4.8	12.4	8	0.7	0.6	8	4.5	0.5
IR	normSe	*Ogg1*$^{+/-}$	Day 59	8	8.4	18.5	8	4.5	4.5	8	5.0	0.4
IR	normSe	*Ogg1*$^{-/-}$	Day 59	6	4.1	6.8	6	0.8	0.4	6	5.0	1.0
IR	lowSe	*Ogg1*$^{+/-}$	Day 59	7	1.0	1.4	7	2.4	4.4	7	4.9	0.8
IR	lowSe	*Ogg1*$^{-/-}$	Day 59	8	1.0	1.0	8	0.6	0.7	8	5.7	1.7
ENU	normSe	*Ogg1*$^{+/-}$	Day 14	2	96.1	17.4	2	23.8	7.1	2	6.3	0.5

S3. Original data for the measurement of micronuclei.

				%MN-RET			%MN-NCE			%RET		
Irradiation	Diet	Genotype	Experimental Day	N	Mean	SD	N	Mean	SD	N	Mean	SD
non-IR	normSe	*Ogg1*$^{+/-}$	Day 45	8	0.47	0.05	8	0.31	0.03	8	2.56	0.27
non-IR	normSe	*Ogg1*$^{-/-}$	Day 45	8	0.53	0.10	8	0.31	0.04	8	2.42	0.46
non-IR	lowSe	*Ogg1*$^{+/-}$	Day 45	8	0.46	0.10	8	0.31	0.03	8	3.27	1.54
non-IR	lowSe	*Ogg1*$^{-/-}$	Day 45	8	0.46	0.05	8	0.30	0.02	8	2.82	0.66
IR	normSe	*Ogg1*$^{+/-}$	Day 45	8	0.66	0.08	8	0.40	0.05	8	2.40	0.38
IR	normSe	*Ogg1*$^{-/-}$	Day 45	8	0.71	0.20	8	0.39	0.05	8	2.11	0.53
IR	lowSe	*Ogg1*$^{+/-}$	Day 45	8	0.73	0.08	8	0.40	0.02	8	2.59	0.42
IR	lowSe	*Ogg1*$^{-/-}$	Day 45	8	0.69	0.08	8	0.41	0.02	8	2.88	0.53
